# Supplementary material for: Adjustment of light-responsive NADP dynamics in chloroplasts by stromal pH
Source: Nat Commun. 2023 Nov 6;14:7148. doi: 10.1038/s41467-023-42995-9 (PMC10628217; doi:10.1038/s41467-023-42995-9)
Supplement: Supplementary file 1 — Supplementary Information [file 41467_2023_42995_MOESM1_ESM.pdf]

Supplementary Figures

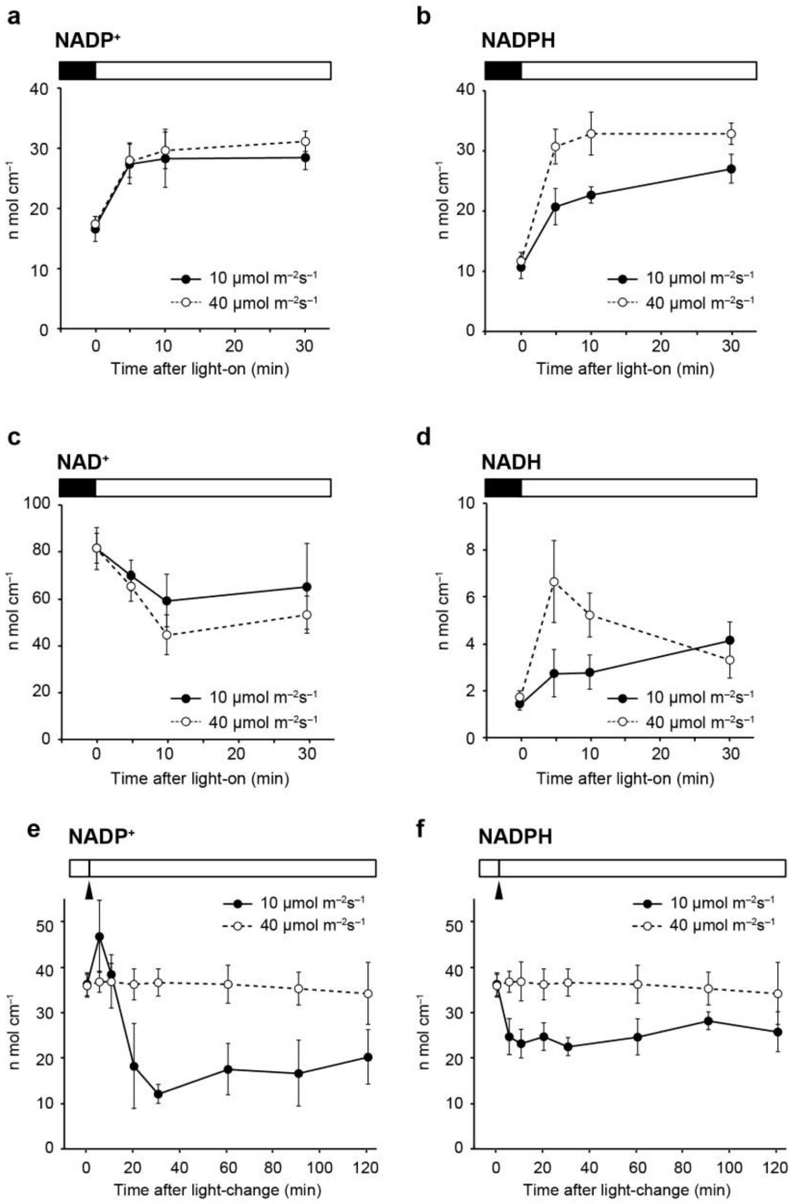

Supplementary Figure 1. Light-dependent response of $NADP^+$, NADPH, NAD^+, and NADH

a–d, Response to light exposure: $NADP^+$ (**a**), NADPH (**b**), NAD^+ (**c**), and NADH (**d**) in *Arabidopsis* leaf discs (biological replicates *n* = 6; data represents mean ± s.d.). The black bar at the top of the graph indicates dark conditions and the white bar indicates light conditions. **e–f**, Response of $NADP^+$ (**e**) and NADPH (**f**) to change in light intensity from 40 µmol m^{-2} s^{-1} to 10 µmol m^{-2} s^{-1} (biological replicates *n* = 6; data represents mean ± s.d.). Leaf discs were acclimated to the dark for 120 min before illumination. The white bar at the top of the graph indicates the light condition, indicating that the light intensity was readjusted at the triangular point after acclimatization at 40 µmol m^{-2} s^{-1}.

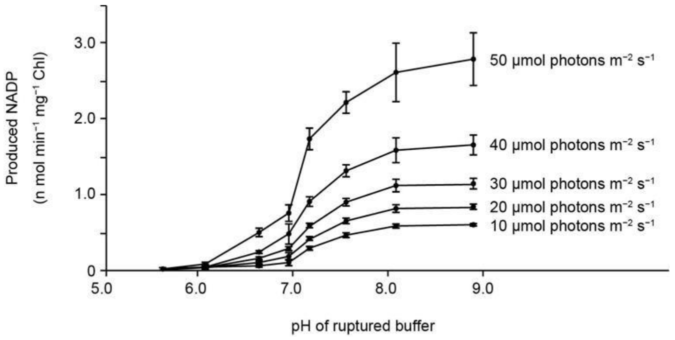

Supplementary Figure 2. NADP synthesis under various light intensity and pH conditions

Isolated chloroplasts were light acclimated for 30 min at the indicated light intensities and ruptured in a buffer containing NAD^+ under various pH conditions. After 30 min of incubation under each light intensity, $NADP^+$ and NADPH were quantified as described in the methods section. NADP synthesis activity was presented as the sum of produced $NADP^+$ and NADPH per min normalized by chlorophyll content (mg) (biological replicates *n* = 4; data represents mean ± s.d.).

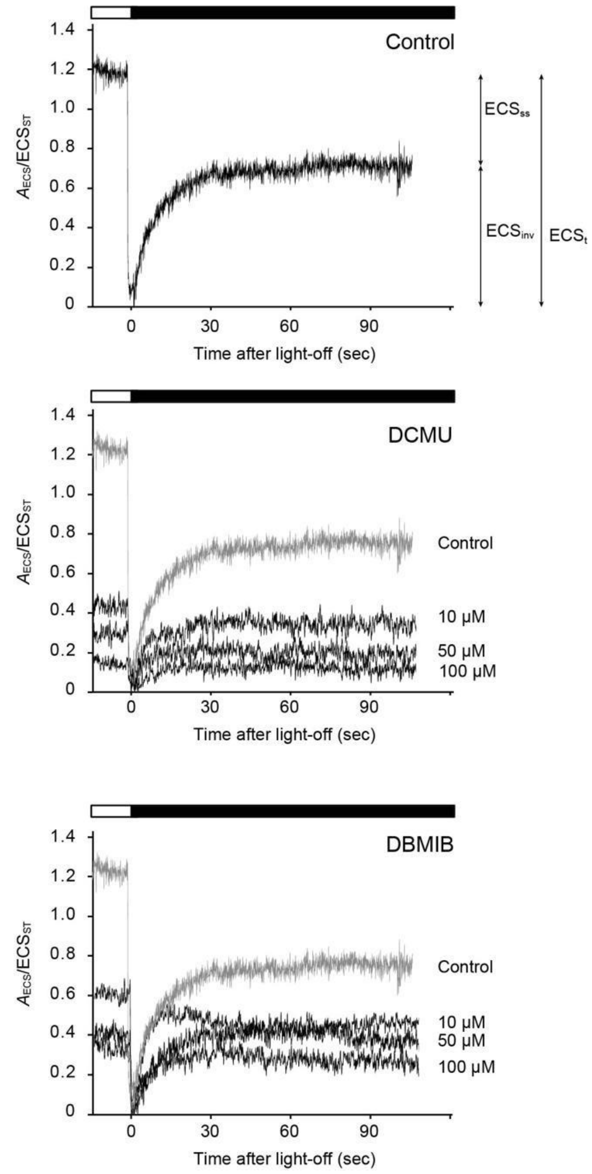

Supplementary Figure 3. Representative data of the *pmf* parsing traces

Leaf discs were illuminated for 3 min with actinic light (AL) at 167 μmol m^{-2} s^{-1}, and then AL was turned off (time 0). Full ECS decay kinetics in the dark was monitored to measure ΔpH (ECS_{inv}) and ΔΨ (ECS_{SS}). The total change in the 515–550 nm absorbances (ΔA_{ECS}) was normalized against the 515–550 nm absorption change induced by single turnover flash (ECS_{ST}). Representative of three independent experiments with similar results was shown. The black bar at the top of the graph indicates dark conditions and the white bar indicates light conditions.

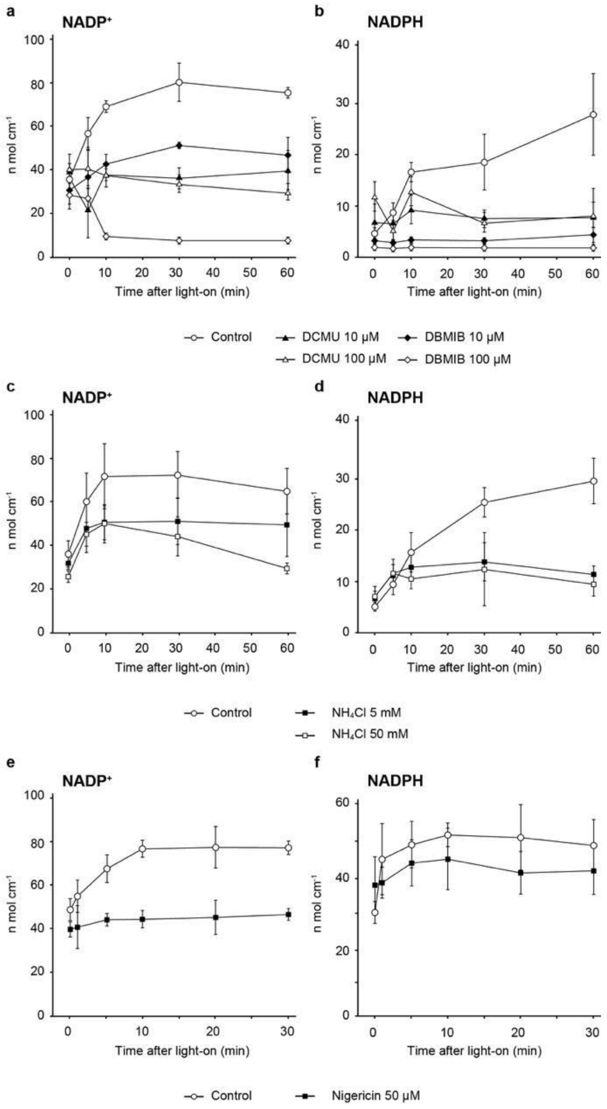

Supplementary Figure 4. Response of NADP+ and NADPH to light in the presence of photochemical inhibitors, ammonium chloride and Nigericin

a–f, Light-dependent behaviours of NADP pool in the presence of photochemical inhibitors (**a, b**), ammonium chloride (**c, d**) and Nigericin (**e, f**) in Col-0. Each panel shows the behaviours of NADP+ (**a, c, e**) and NADPH (**b, d, f**). Leaf discs acclimated to dark for 90 min were subjected to reduced pressure infiltration with the indicated reagent in the dark for 10 min and then illuminated at 70 μmol m^{-2} s^{-1} (biological replicates n = 3 for photochemical inhibitors and n = 6 for ammonium chloride and Nigericin; data represents mean ± s.d.).

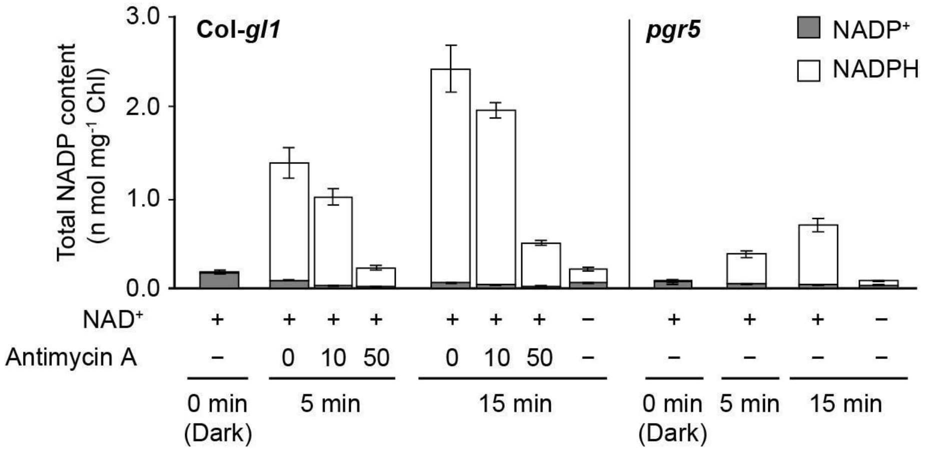

Supplementary Figure 5. Response of NADP pool size to light in the presence of antimycin A

Response of NADP pool size to light in the presence of antimycin A. Dark-acclimated isolated chloroplasts prepared from *Col-gl1* were illuminated for 5 min or 15 min at 10 μmol m^{-2} s^{-1} with 5 mM NAD^+ in the absence or presence of antimycin A. Similarly, dark-acclimated isolated chloroplasts prepared from *pgr5* were also illuminated for 5 min or 15 min at 10 μmol m^{-2} s^{-1} with 5 mM NAD^+ in the absence of antimycin A. Subsequently, $NADP^+$ and NADPH were separately measured and the sum of $NADP^+$ and NADPH was demonstrated as total NADP content in graphs. Data represent mean ± SD (n = 6 biological replicates without Antimycin A and *n* = 3 biological replicates with Antimycin A).

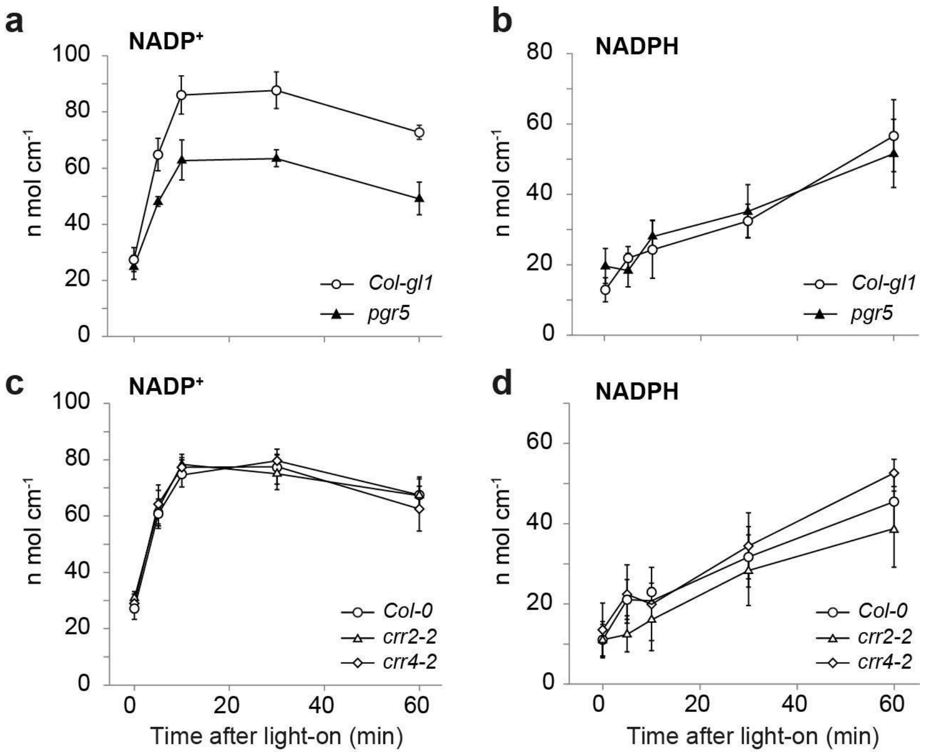

Supplementary Figure 6. Response of $NADP^+$ and NADPH to light in CET mutants

a–d, Light-dependent behaviours of NADP pool in CET mutants: PGR5/PGRL1 pathway (**a, b**), and NDH pathway (**c, d**). Each panel shows the behaviours of $NADP^+$ (**a, c**) and NADPH (**b, d**). Leaf discs prepared from the indicated *Arabidopsis* lines were acclimated to dark for 120 min and then illuminated at 70 μmol m^{-2} s^{-1} (biological replicates $n = 3 \pm$ s.d.).

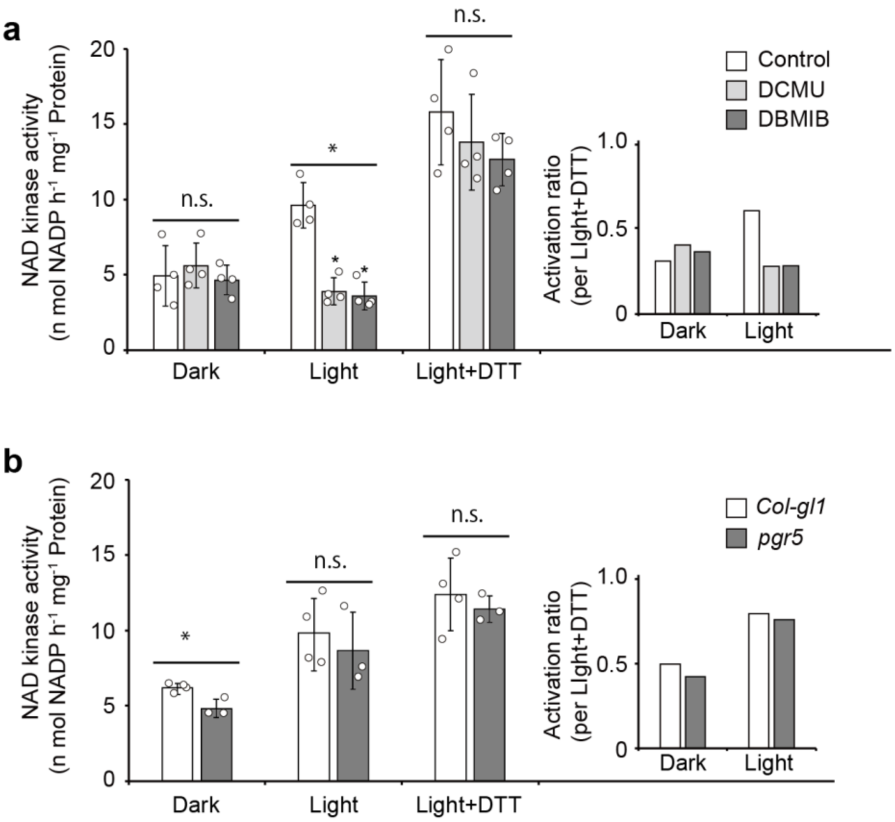

Supplementary Figure 7. Light-dependent activation of NAD$^+$ phosphorylation

a, Light-dependent activation of NAD$^+$ phosphorylation in the presence of photochemical inhibitors (biological replicates *n* = 4; data represents mean ± s.d.). Protein extracts were prepared from leaf discs incubated at 70 μmol m^{-2} s^{-1} for 30 min with 0.1% DMSO (white bars), DCMU (light grey bars) or DBMIB (grey bars) in the presence or absence of DTT. Asterisks indicate statistically significant differences between Control, DCMU and DBMIB (one-way ANOVA): $p = 5.732\times10^{-5}$ in Light. n.s. means statistically no significance: $p = 0.681$ in Dark and $p = 0.344$ in Light + DTT. **b,** Comparison of light-dependent activation of NAD$^+$ phosphorylation between *Col-gl1* (white bars) and *pgr5* (grey bars) (biological replicates *n* = 4 in *Col-gl1* and *n* = 3 in *pgr5*; data represents mean ± s.d.). Asterisks indicate statistically significant differences between *Col-gl1* and *pgr5* (*t*-test): $p = 0.010$ in Dark. n.s. means statistically no significance: $p = 0.541$ in Light and $p = 0.534$ in in Light + DTT. Protein extracts were prepared form leaf discs incubated at 70 μmol m^{-2} s^{-1} for 30 min with or without DTT. All assays were performed using 2 μg of extracted protein and produced NADP$^+$ was measured with NADP-Glo assay. Activation ratios are plotted in the inset.

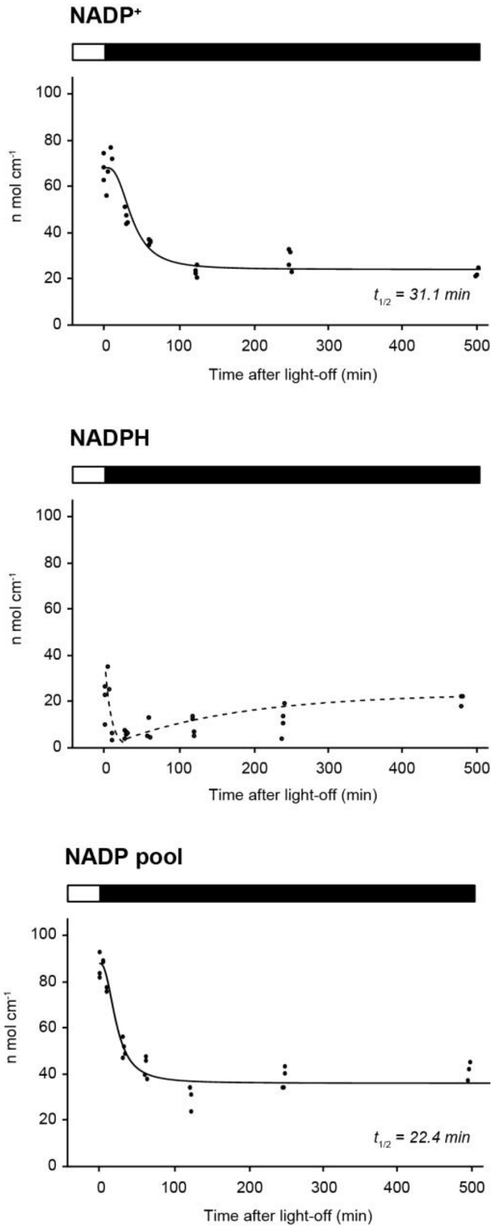

Supplementary Figure 8. Response of $NADP^+$ and NADPH to dark

Leaf discs prepared from Col-0 were acclimated to light at 70 μmol m^{-2} s^{-1} for 120 min and then immediately placed in the shade so that the light intensity was less than 10 μmol m^{-2} s^{-1} for the indicated durations. The time at which the light-regulated amount was halved ($t_{1/2}$) was estimated from the fitting curve drawn using ImageJ2 (https://imagej.net/). The black bar at the top of the graph indicates dark conditions and the white bar indicates light conditions.

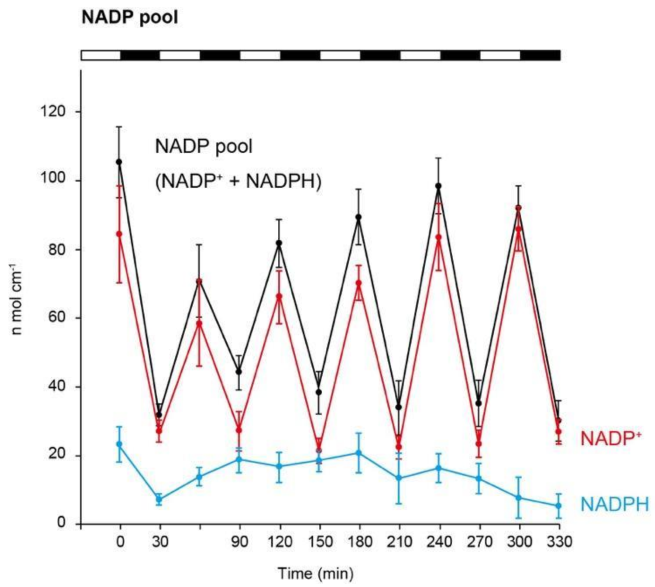

Supplementary Figure 9. Fluctuation of NADP pool size in response to light and dark

Responses of $NADP^+$, NADPH, and NADP pool size to a 30 min light/dark cycle. Data are presented as mean ± s.d. (n = 6 biological replicates). Leaf discs were acclimated to light at 70 μmol m^{-2} s^{-1} for 120 min before the first dark treatment. The black bar at the top of the graph indicates dark conditions and the white bar indicates light conditions.

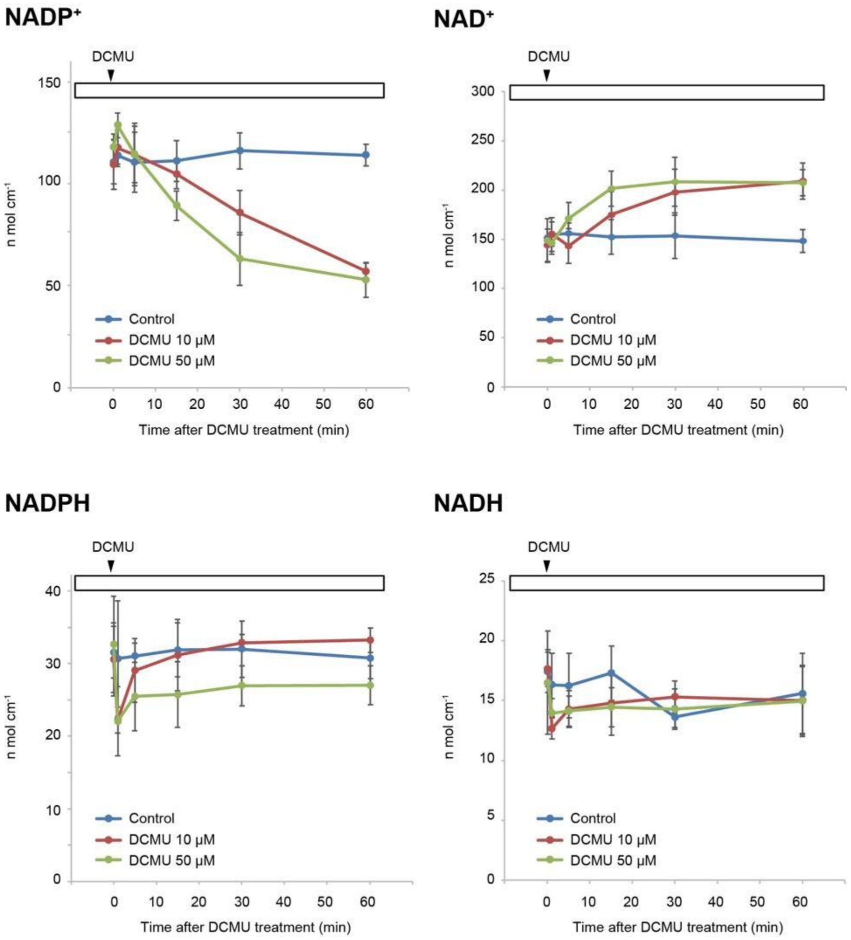

Supplementary Figure 10. Responses of $NADP^+$, NADPH, NAD^+, and NADH to DCMU under light conditions

Leaf discs prepared from Col-0 were acclimated to light at 70 μmol m^{-2} s^{-1} for 120 min and then quickly immersed in the DCMU solution and immersed for the indicated durations (biological replicates n = 6; data represents mean ± s.d.). The white bar at the top of the graph indicates the light condition, indicating that DCMU was applied at the triangular point.

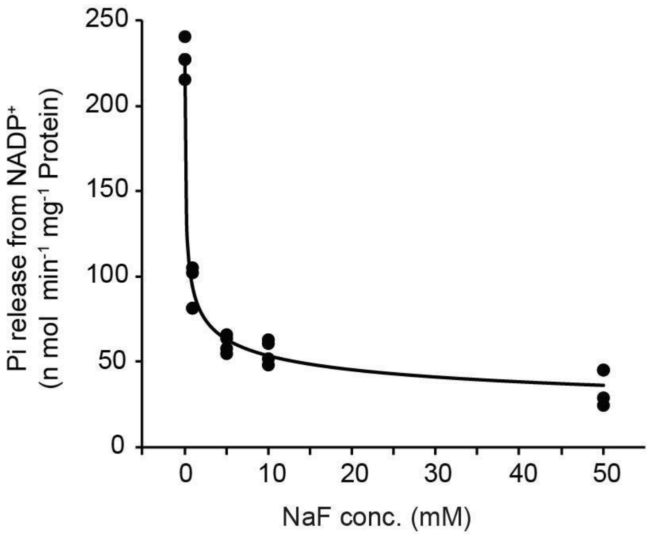

Supplementary Figure 11. Sensitivity of $NADP^+$ phosphatase activity to sodium fluoride

NADPP activity with increasing amounts of sodium fluoride (NaF). All assays were performed using 1 μg of extracted protein from the dark-acclimated leaf discs.

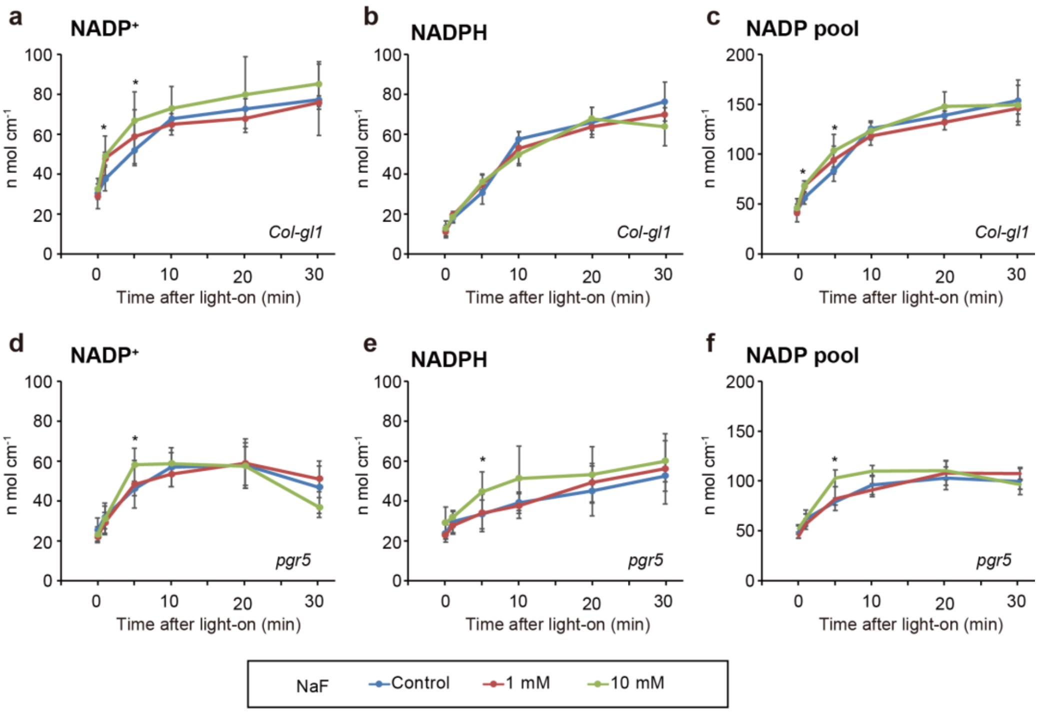

Supplementary Figure 12. Responses of $NADP^+$, NADPH, and NADP pool size to light in the presence of phosphatase inhibitor sodium fluoride

a–f, Response of $NADP^+$ (**a, d**), NADPH (**b, e**), and NADP pool size (**c, f**) to light in *Col-gl1* (wildtype) (**a-c**) and *pgr5* (**d–f**) (biological replicates, n = 6; data represents mean ± s.d.). Leaf discs acclimated to dark for 90 min were subjected to reduced pressure infiltration with 1 mM or 10 mM sodium fluoride (NaF) in the dark for 10 min. The leaf discs were then illuminated at 70 μmol m^{-2} s^{-1}. Asterisks indicate statistically significant differences between Control and 10 mM NaF ($p < 0.05$, t-test).

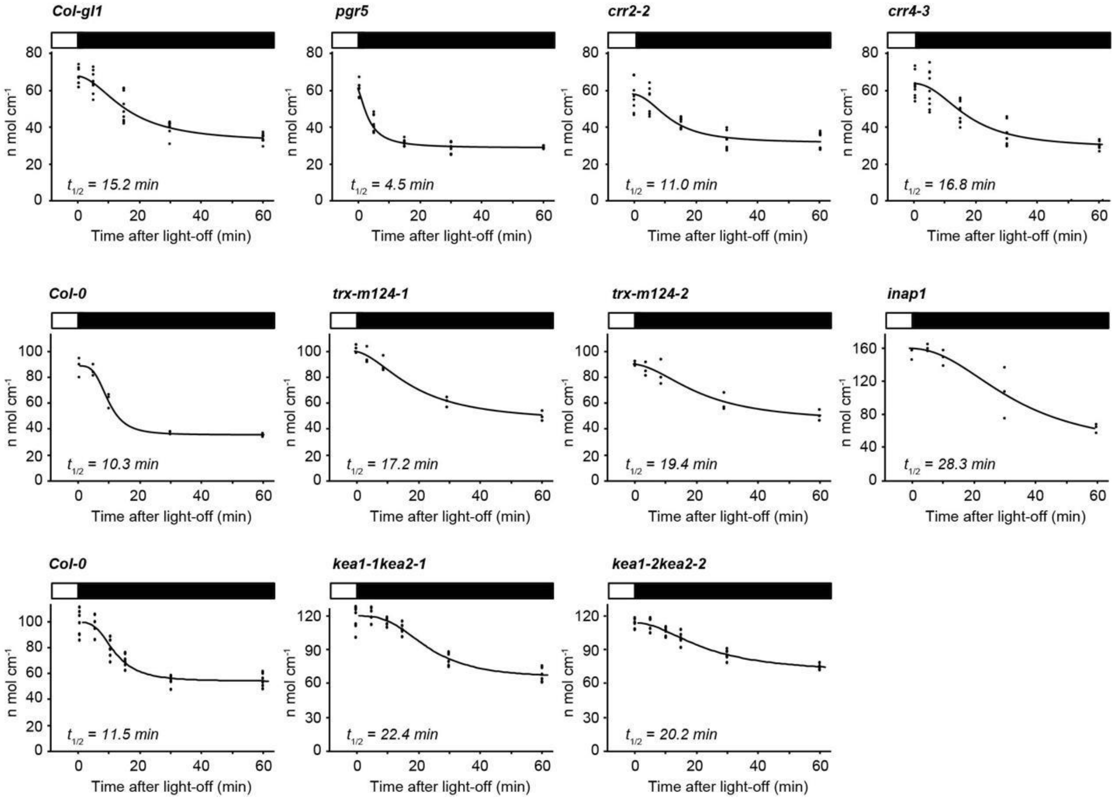

Supplementary Figure 13. Dark-induced decrease of NADP pool in CET mutants and *kea1kea2* mutants

Leaf discs prepared from indicated *Arabidopsis* lines were acclimated to light at 70 µmol m^{-2} s^{-1} for 120 min and then immediately placed in the shade so that the light intensity was less than 10 µmol m^{-2} s^{-1} for the indicated durations (biological replicates *n* = 9 for *Col-gl1*, *pgr5*, *crr2-2*, *crr4-3*, *n* = 3 for *Col-0*, *inap1*, *trxm124-1*, *trxm124-2*, *n* = 6 for *Col-0*, *kea1-1kea2-1*, *kea1-2kea2-2*; data represents mean ± s.d.). The time at which the light-regulated amount was halved ($t_{1/2}$) was estimated from the fitting curve drawn using ImageJ2 (https://imagej.net/). The black bar at the top of the graph indicates dark conditions and the white bar indicates light conditions.

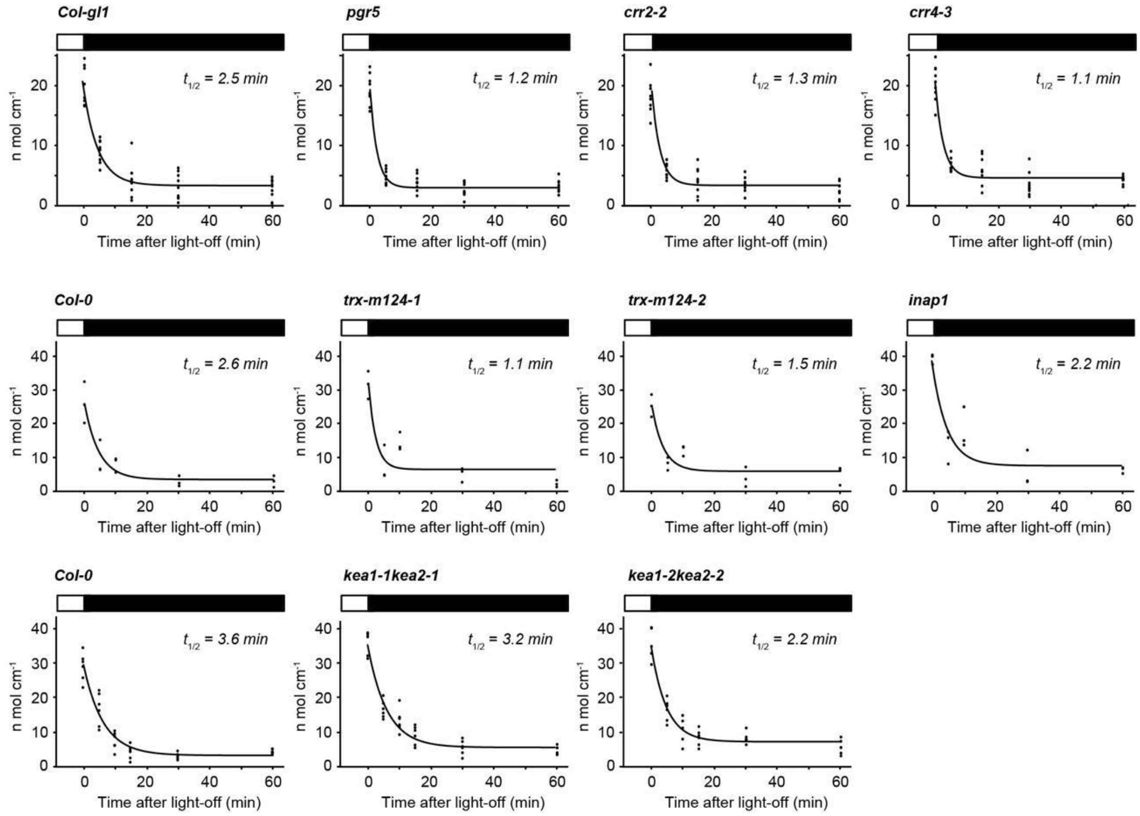

Supplementary Figure 14. Dark-induced decrease of NADPH in CET mutants and *kea1kea2* mutants

Leaf discs prepared from indicated *Arabidopsis* lines were acclimated to light at 70 µmol m^{-2} s^{-1} for 120 min and then immediately placed in the shade so that the light intensity was less than 10 µmol m^{-2} s^{-1} for the indicated durations (biological replicates *n* = 9 for *Col-gl1*, *pgr5*, *crr2-2*, *crr4-3*, *n* = 3 for *Col-0, inap1*, *trxm124-1*, *trxm124-2*, *n* = 6 for *Col-0, kea1-1kea2-1*, *kea1-2kea2-2*; data represents mean ± s.d.). The time at which the light-regulated amount was halved ($t_{1/2}$) was estimated from the fitting curve drawn using ImageJ2 (https://imagej.net/). The black bar at the top of the graph indicates dark conditions and the white bar indicates light conditions.

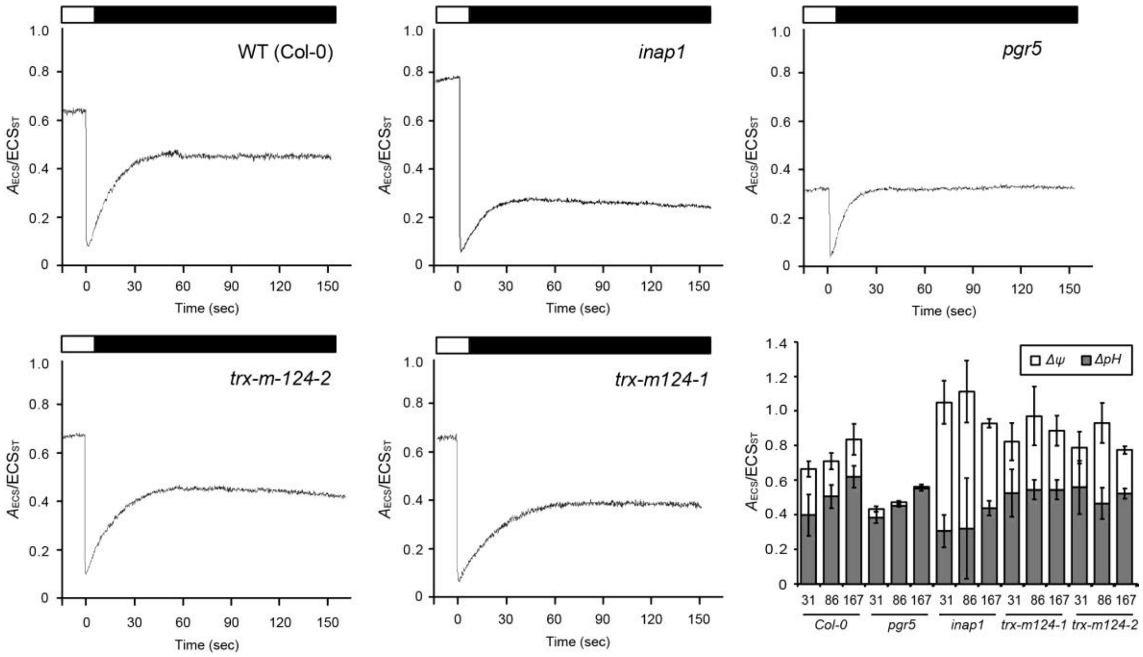

Supplementary Figure 15. Components of *pmf* and representative parsing traces

Leaf discs were illuminated for 3 min with actinic light (AL) at the indicated light intensity, and then AL was turned off (Time 0). Full ECS decay kinetics in the dark was monitored to measure ΔpH (ECS_{inv}) and ΔΨ (ECS_{SS}). The total change in the 515–550 nm absorbances (ΔA_{ECS}) was normalized against the 515–550 nm absorption change induced by single turnover flash (ECS_{ST}). The black bar at the top of the graph indicates dark conditions and the white bar indicates light conditions. Data are presented as mean ± s.d. (n = 3 biological replicates).

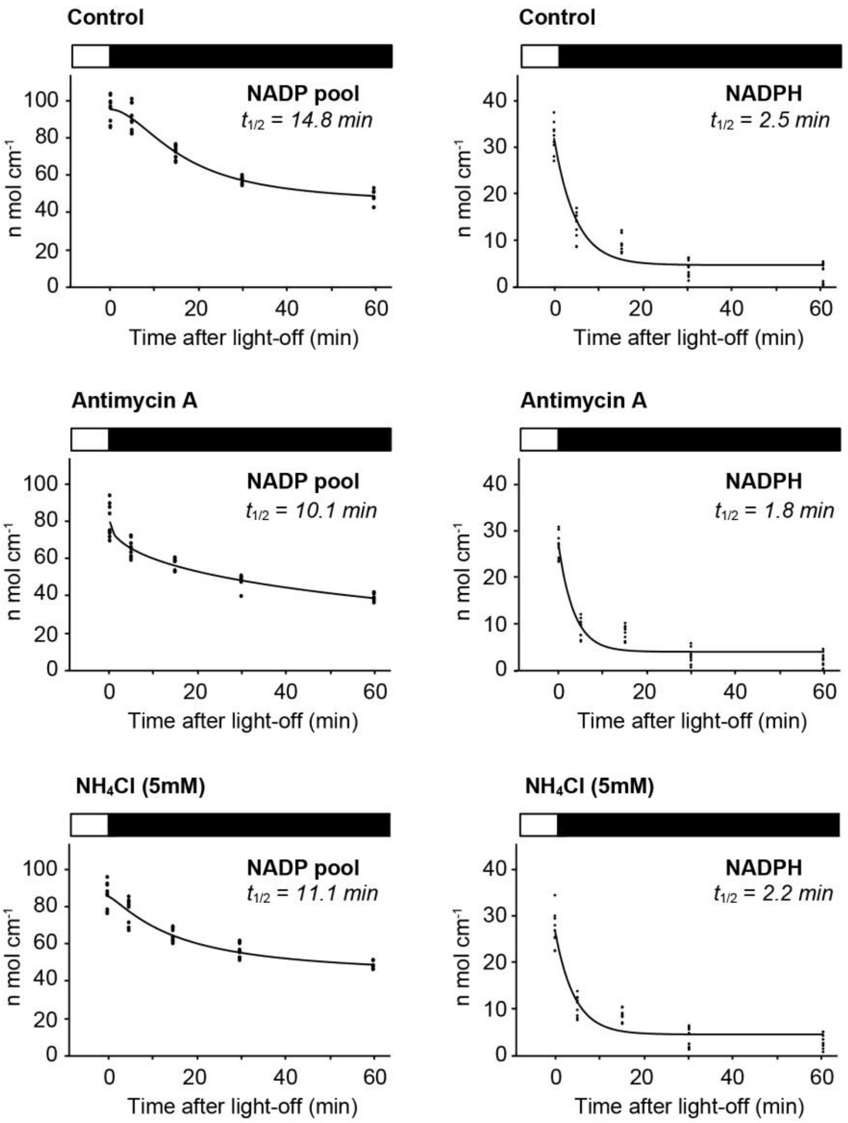

Supplementary Figure 16. Response of NADP pool size and NADPH to antimycin A and ammonium chloride under dark

Leaf discs prepared from Col-0 were acclimated to light at 70 μmol m^{-2} s^{-1} for 115 min floated on 1% (v/v) ethanol (Control). Dark-response experiments were initiated immediately after the addition of an equal volume of solution containing 10 mM ammonium chloride, or 100 μM Antimycin A to 1% (v/v) ethanol in which the leaf discs were floating under light. They were immediately placed in the shade so that the light intensity was less than 10 μmol m^{-2} s^{-1} for the indicated duration (biological replicates n = 6 ± s.d.). The time at which the light-regulated amount was halved ($t_{1/2}$) was estimated from the fitting curve drawn using ImageJ2 (https://imagej.net/). The black bar at the top of the graph indicates dark conditions and the white bar indicates light conditions.
